# Natural and human-impacted diversity of bryophytes along an elevational gradient on an oceanic island (La Palma, Canarias)

**DOI:** 10.1371/journal.pone.0213823

**Published:** 2019-04-03

**Authors:** Raquel Hernández-Hernández, Jürgen Kluge, Claudine Ah-Peng, Juana María González-Mancebo

**Affiliations:** 1 Departamento de Botánica, Ecología y Fisiología Vegetal, Plant Conservation and Biogeography Group, Universidad de La Laguna, C/Astrofísico Francisco Sánchez, s/n. La Laguna, Islas Canarias, España; 2 Philipps-Universität Marburg, Dept. Geographie, Marburg, Germany; 3 Université de La Réunion, UMR PVBMT, France; Sikkim University, INDIA

## Abstract

Bryophytes have been proposed as ideal indicators of ecosystem change, because they are important components of forest integrity, and considerable research indicates that some groups are sensitive to the changes associated with specific human disturbances. Bryophyte richness and abundance have been found to vary predictably along elevational gradients, but the role of human impacts on these distribution patterns remains unclear. The aim of this study is to explore the impact of human disturbance on the elevational patterns of bryophyte diversity, along an elevational gradient. Along the gradient we collected three datasets in the following sites: preserved (P), forest track roadsides (R) and disturbed by agriculture/silviculture practices (D). Two survey plots of 100 m^2^ were established at every 200 m elevational step for each sites P, R, D, and in each plot bryophytes were sampled in a stratified manner. At each plot we recorded all species on available substrates and estimated their percentage cover. Our results showed that species number did not differ among studied sites, but that species diversity pattern differs among the three gradient types and species life strategy composition along the elevational gradient showed a clear response to the disturbance of mature communities. We conclude that human impact has strongly changed the elevational pattern of diversity, and that these changes vary depending on the ecological and taxonomical group considered.

## Introduction

Disturbances are defined as changes in the biotic or abiotic environment that alter the structure and dynamics of ecosystems [1]. Whether natural or human-induced, disturbances play an important role in shaping global vegetation [2,3,4].

Since prehistoric times humans have shown a great ability to colonise new environments and drastically modify them [5]. No ecosystem on Earth’s surface is free of pervasive human influence [5]. There is a growing impulse to estimate the human effects in the future (e.g. [6,7,8]). However, the importance of the past and current human influence is not always recognized. This factor is frequently underestimated or ignored [9] in ecological studies, and thus, described patterns of species distribution might be misunderstood. For example, changes that are attributed to environmental filters may be instead due to past or present human activities. Several studies provide evidence that the effects of past human disturbance remain in the ecosystems even after long periods of natural regeneration of the forest, as has been observed for plants [10], butterflies [11] and small mammals [12].

Species richness and elevation often show complex relationships, which are dependent on the taxonomic group and locality considered [13]. Biodiversity patterns along elevational gradients have mostly been related with climatic conditions, area of elevational belts and thus size of source populations, environmental heterogeneity and ecological isolation (e.g. [14,15,16]). However, spatial variation in natural resources along the gradient is usually related to human disturbances, which may modify dispersal, colonization and species distributions. Studies on species diversity along elevational gradients date back to the origin of biogeography [17]. However, the relationship between human impact and diversity along elevational gradients has seldom been considered [18]. In a review of the literature on species–elevation relationships, Nogués-Bravo et al. [9], found that a high number of the elevational analyses contained disturbed areas. However, few of these studies included a measure of human impact as a potential predictor.

Bryophytes are the most diverse group of land plants after the flowering plants [19]. They are distributed in almost every terrestrial ecosystem in the globe, so they are ideal candidates for latitudinal and altitudinal studies [20]. Due to their poikilohydric nature, bryophytes are very sensitive to environmental changes and can be used as bioindicators of environmental and microclimatic conditions [21]. Bryophytes have been found to be affected at both habitat and landscape-scale by human disturbances [22,23,24]. The effects of stand replacing plantations and clear cuts has been previously addressed in the Canary Islands laurel forests [23,25,26], however, the effects of other types of disturbance on bryophyte communities remains unknown.

The importance of human impact along elevational gradients has been recently observed in a study with vascular plants in La Palma island, where influence of past disturbances was found to affect processes such as speciation and invasion along the gradient [27]. However, few studies include the anthropogenic factor as a direct variable in studies of species distribution. Here we study diversity patterns of bryophytes along a complete elevational gradient from sea level to high mountain peaks in the Canary Islands (0–2200 m). Our general aim is to explore the impact of human disturbances on the elevational patterns of bryophyte diversity. We ask the following specific questions: 1) What is the diversity pattern, life strategy composition, substrate affinities and species distributions along the elevational gradient? 2) Do diversity patterns differ in preserved (undisturbed) and disturbed sites?

## Material and methods

### Study area

**Fig 1:**
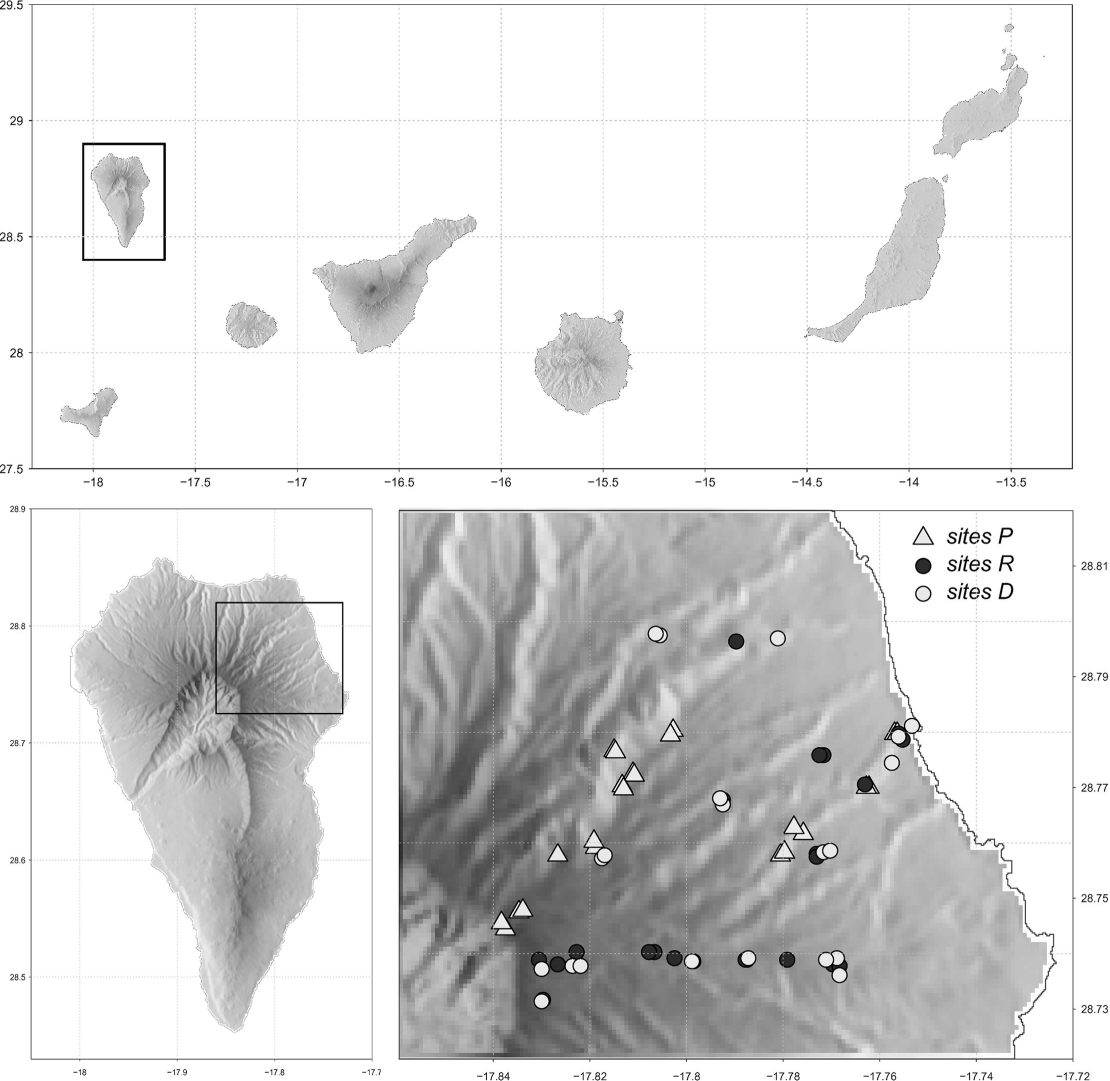
Location of the study area in La Palma Island and position of the three types of sites along the elevational transect.

**Fig 2:**
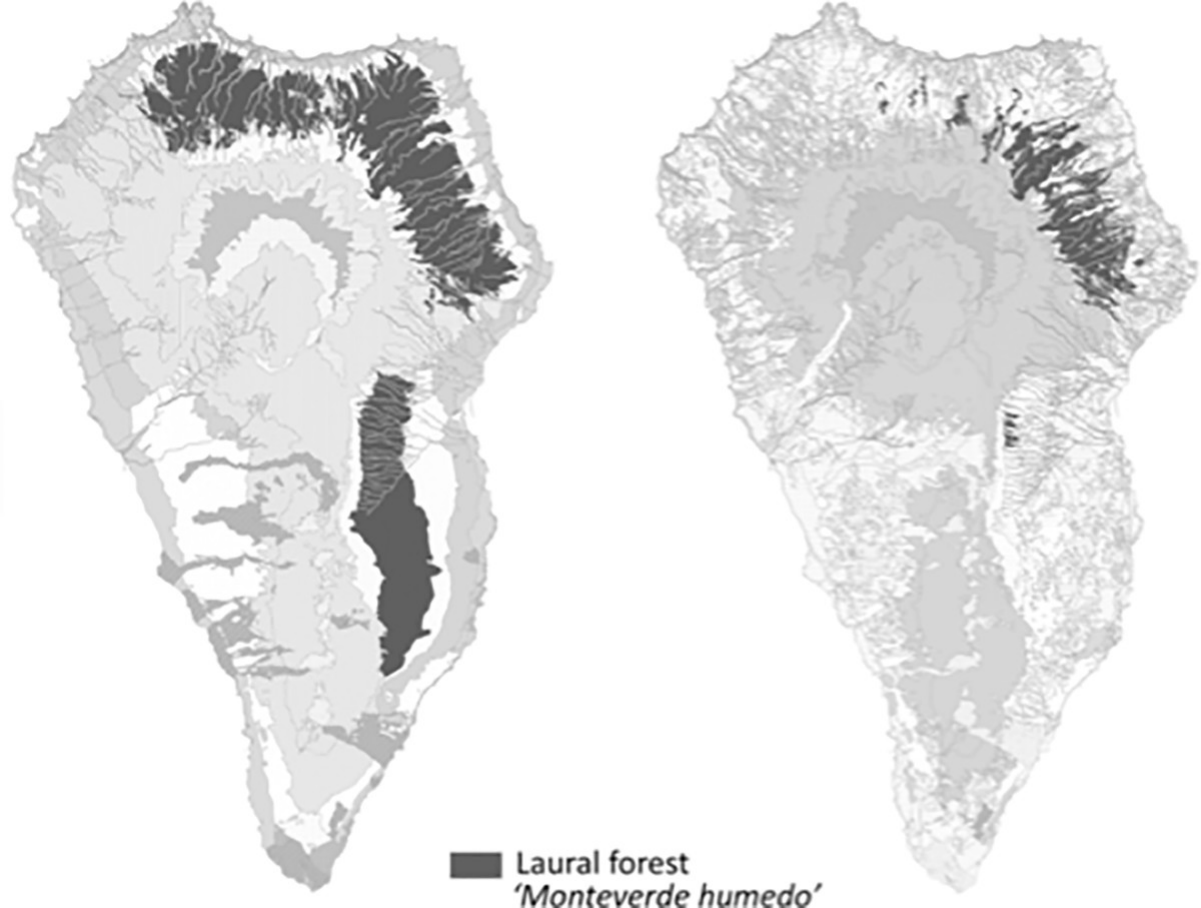
Potential (left) and actual (right) areas of occupancy of the laurel forest in La Palma area. Data based on Del Arco et al. 2006 [31].

The study was conducted on La Palma Island (Canary Islands). The Canary Islands are situated at a latitude of about 28°N and longitude between 13° and 18°W (Fig 1) and belong to the Macaronesian floristic region [28]. This archipelago represents one of the most diverse areas in the Mediterranean basin hot-spot of biodiversity [28,29]. La Palma Island is one of the occidental and youngest islands of the archipelago, with an estimated age of 1.7 Myr [30]. It has a relatively small area (706 km^2^), but it is the second highest island (2396 m a.s.l), thus comprising steep ecological gradients and a large variety of bioclimatic belts. The north and east slopes of the islands are especially exposed to the trade winds below 1500 m a.s.l, which make them extremely humid. The lowland areas of the island (0–200 m a.s.l.) are occupied by sub-desert, shrubby vegetation, dominated by species of the genus *Euphorbia*. Above the shrubland, at 200–400 m a.s.l., the potential vegetation is a thermophyllous woodland (Dry *Apollonias barbujana* forest), but this formation has been almost destroyed due to human activities on all islands of the archipelago [31]. The next higher zone is the potential range of the endemic laurel forest (300–1400 m a.s.l.). Much of this vegetation type has been destroyed in La Palma, but there are some small areas with mature and well-preserved forest stands (Fig 2). From 1500 to 2000 m a.s.l. there is the endemic pine (*Pinus canariensis*) forest, with a transition zone of 1200 and 1500 m.a.s.l. with elements both of laurel and pine forests (mixed Pine forest). Above the timberline (>2000 m) a subalpine shrub vegetation follows, dominated by *Adenocarpus foliolosus*.

### Study sites

We established an elevational transect on the north-east slopes of the island,where we studied three different types of sites: preserved (P sites), roadsides (R sites) and disturbed by agricultural/sylvicultural practices (D sites) (Fig 1, Table 1). The transect started at the coast (40 ma.s.l) and ended at the highest elevation possible (2200 m.a.s.l for P sites and 2000 m.a.s.l for D and R sites). The P sites were located at the best preserved areas (Table 1), which are not pristine forests, but correspond to the least disturbed forests present on the island. The R sites are established in forest tracks that traverse well preserved areas. In these tracks, trees are initially cut for the road installation, and due to the high degree of light exposure following the cutting, herbaceous and shrubby species largely dominate the track margins, although young trees are sometimes present. In addition, borders are periodically cut for the maintenance of the roads. The D sites included areas where the original vegetation was removed and replaced by fruit tree plantations in the lower elevations, e.g., banana, papaya, avocado and timber tree plantations in the middle and upper elevations.

**Table 1:** Description of the number of plots and the type of vegetation at each elevational belt at each site.

Elevational range	Plots per site	Potential vegetation (P sites)	Vegetation on R sites	Vegetation on D sites
0–200	2	Euphorbia shrubland	Roadsides with shrubs and young trees	Fruit trees plantations
200–400	2	Dry *Apollonias barbujana* forest	Roadsides with shrubs and young trees	Fruit trees plantations
400–1200	10	Laurel forests *s*.*l*.	Roadsides with shrubs and young trees	Fruit trees plantations and timber extraction
1400–1800	6	*Pinus canariensis* forests	Roadsides with shrubs and young trees	Tree plantation for timber extraction and prescribed burning
2000–2200	4	Subalpine shrub vegetation	Roadsides with shrubs and young trees	Prescribed burning and livestock

The fieldwork was conducted from April 2012 to April 2013. The sampling design followed BRYOLAT methodology [32,33,34,35]. Within each site we studied: plots, subplots and microplots. Plots were established approximately at each 200 m, thus resulting in 12 and 11 elevational levels respectively for P and for R and D. Two plots of 10 m x 10 m were established at each elevational level. Plots in P sites included only well-preserved laurel forests. Plots in site R included the total track area as well as 1 m. of the track margin, in which vegetation was present. Plots in site D were selected where human activity was the most pronounced at each elevational level. These were either fruit plantations or sites managed for forestry.

### Sampling method

Within plots, we randomly selected three subplots of 2x2 m and within each one, bryophyte species were recorded in three microplots of 10x5 cm on each substrate present (soil, rocks, humus and decaying wood; epiphyllous species were absent). Epiphytic bryophytes were also recorded, but in this case 3 individuals of each one of the tree species present in the plot were selected, and 3 microplots were sampled on each tree trunk at three different heights (TA: 0–50 cm, TB: 50–100 cm; TC: 100–200 cm). Number of trees sampled on each site varied from 46 on sites P, to 22 on sites D and no tree was sampled on transect R, due to an absence of epiphytes on the trees that were present on the margins of the tracks. At each microplot, bryophyte samples were first identified in the field to estimate relative cover of each species on each microplot, and then taken to the lab for confirmation. All samples are deposited in the herbarium TFC-Bry of La Laguna University (Tenerife, Canary Islands). Nomenclature follows Losada-Lima et al, 2010 [36]. As a result of this stratified sampling, we sampled a total of 68 microplots along the elevational gradient (P sites: 24 microplots; R and D: each 22 microplots), and within these a total of 194 subplots (P: 70 subplots, R: 65, D: 59). The number of subplots sampled in the different transects varied because bryophytes were not always present on every substrate or the particular substrate was absent. For analyses, data from microplots on each substrate were pooled to the level of these subplots by averaging mean cover values for each species. At each plot, we took a detailed description of the vegetation, recording all vascular species present, their relative cover and whether they were herbs, shrubs or trees. In addition, the following potentially informative environmental variables were recorded: elevation, canopy cover, percentage of rocks, percentage of litter layer, and percentage of bare soil.

### Data analysis

Bryophytes were assigned to categories reflecting their life-history strategy (Table 2). Life strategy classification followed Düring (1979) [37]. For the analyses, specimens of the division Anthocerotophyta were included together with Marchantiophyta, due to their low number of species and occurrence along the gradient. Total number of species was calculated for sites P, R and D. For analyses, microplot data for each substrate was pooled within subplots by averaging mean cover values for each species.

**Table 2:** Life strategy classification based on Düring (1979) [37].

Life strategy	Spore size	Spore persistence	Sporophyte production	Life span
Fugitives	<20 μm	very persistent and long lived	High	Short
Colonists	<20 μm	very persistent	High	Moderately short
Annual shuttles	>20 μm	Several years	High	Short
Short-lived shuttles	>20 μm	Several years	Rather high	Few years
Long-lived shuttles	>20 μm	Short	Moderate	Long
Perennial stayers	<20 μm	Variable	Low	Many years (>20)

We calculated number and percentage cover of mosses, liverworts (with regarding of families) in P, R and D sites and also percentage of life forms in each site type. To highlight elevational trends of species richness, we applied non-parametric locally weighted scatterplot smoothing by using function *lowess* in R (gplots package). It fits trendlines to data subsets in order to produce a smoothed curve making the species richness trend in the figures directly visible.

Separately, for each of the different sites (P, R, D) we used Indicator Species Analysis [38] which weights relative frequencies and relative cover of species within and between each site to search for important diagnostic species. The analysis determines both fidelity (restriction to a site or group of sites considered) and consistency of species (consistent species occurrence among sites within site groups), providing a statistic (IndVal) and an associated *p*-value. Only species significant at the *p* < 0.05 level were selected as indicator species. Indicator species were investigated for elevational belts based on the main type of potential vegetation (Table 1). To analyse elevational range distributions of colonist and perennial species, we took the minimum and maximum elevational range of the respective species, and used the median as the range centre to account for accidental outliers.

In order to assess vegetation similarity and heterogeneity between plots and gradients, we performed ordination techniques (Detrended Correspondence Analysis) based on Bray-Curtis similarity index, with subplots as cases and mean cover of species as a variable. Cover of each species at each microplot was pooled together to obtain the mean value for subplot, for species in every substrate. In order to reduce noise in the dataset, we downweighted rare species. Environmental characteristics recorded in the sampling sites (see methods section) were fitted to the ordination result in order to assess their possible contribution to community assemblage patterns. To assess the separation of sites and vegetation belts within the ordination pattern, we fitted the respective variable onto the ordination scores with 999 permutations, which provides a goodness-of-fit r^2^ and significance p, indicating whether the score centroids are significantly separated. All analyses were performed with the statistical platform R [39] and packages vegan [40] and labdsv [41].

## Results

Along the three site types a total of 178 species were recorded (131 mosses, 44 liverworts and 3 hornworts), belonging to 94 genera and 52 families. Pottiaceae (37 species) was the most common family, followed by Brachytheciaceae (15 species) and Bryaceae (12 species) (See S1 Table). Regarding each considered transect, for the P sites we found a total of 118 species (86 mosses and 32 liverworts), with Pottiaceae and Brachyteciaceae being the most abundant families. One hundred and ten species were recorded in R sites (76 mosses, 31 liverworts and 3 hornworts), with Pottiaceae as the most common family, followed by Bryaceae. In D sites, 99 species were found (69 mosses, 29 liverworts and one hornwort) and Pottiaceae was again the most common family encountered followed in this case by Grimmiaceae. Regarding life strategies, colonists were the most abundant group: 60 species (50%) in P sites; 69 species (63%) in R sites; and 61 species (61%) in D sites. Colonists were followed by perennials: 34 species (28%) in P; 18 species (16%) in R; and 20 species (20%) in D. The other categories were found at a lesser extent in all sites. Among the studied sites some interesting macaronesian endemic species were recorded, such as *Homalothecium mandonii*, *Leptodon longisetus*, and *Exsertotheca intermedia*, all found exclusively in the plots of P sites.

**Fig 3:**
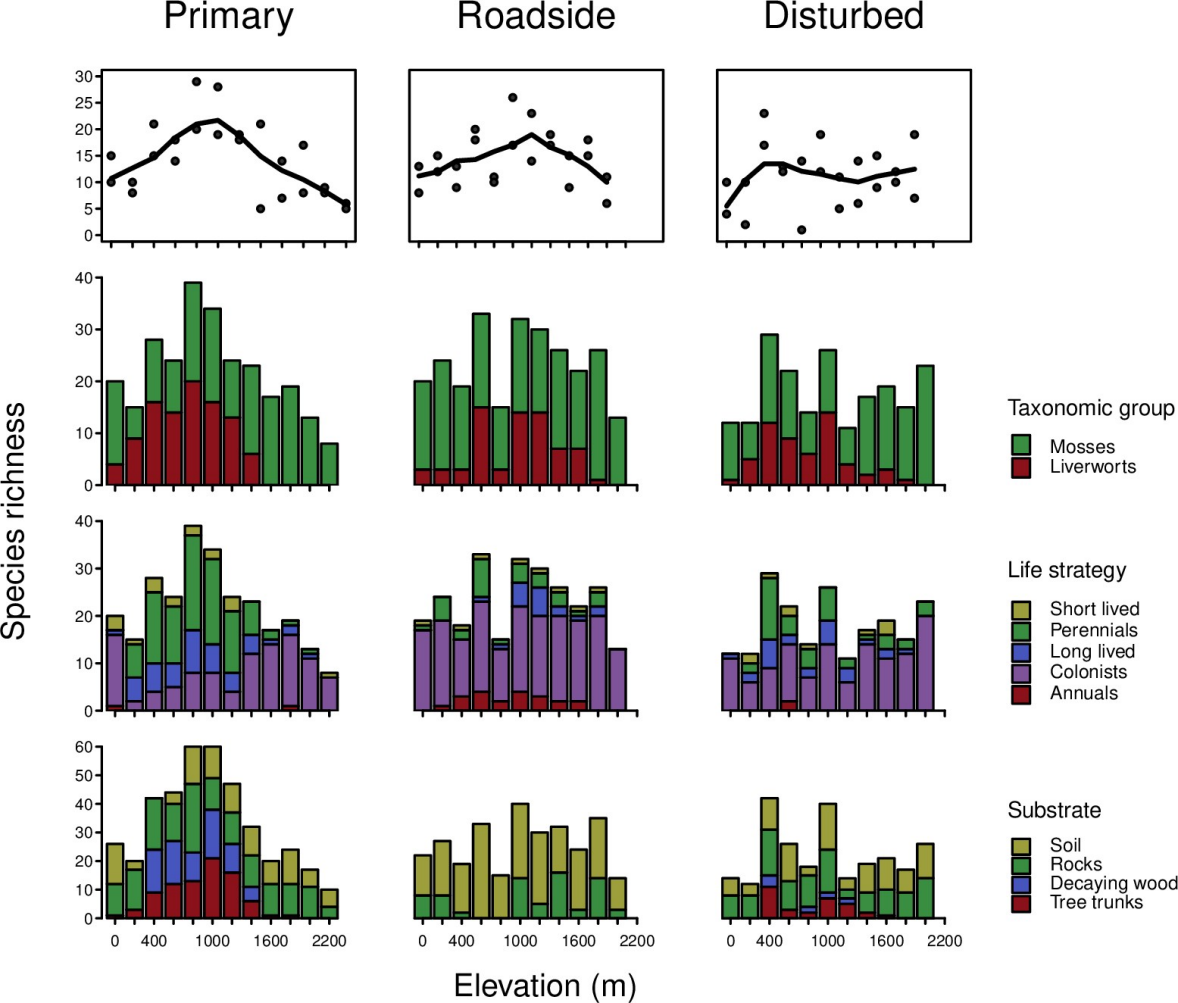
Elevational patterns of species richness in every plot. Trend lines set by distance weighted least square smoothing using *LOWESS* function in R. Bar plots indicate proportion of each taxonomic group, life strategy and substrate type at each elevational band. Number of species along y-axis is different when considering substrate as one species can be present in several substrates, thus total number of species considered increases.

Elevational patterns of species richness showed differences across all three sites types (Fig 3). Distribution of species richness in well preserved vegetation (P sites) showed a hump-shaped relationship with elevation, with maximum species richness at mid-elevation plots at about 1000 m, thus corresponding to the laurel forest. R sites also showed a hump-shaped pattern, but not as clear as in P sites, and in this case the maximum species richness shifted towards higher elevations. In D sites, the hump-shaped pattern was replaced by an increase of species richness up to 400 m a.s.l. followed by a plateau above with a more or less constant number of species until the end of the transect.

Regarding taxonomic groups in P sites, the number of moss species was higher in the lower and upper parts of the gradient, while at mid-elevation, liverworts had a more pronounced contribution (Fig 3). In R sites, mosses were more abundant than liverworts along the entire gradient. The number of mosses was more or less constant, while liverworts showed a hump-shaped pattern (except at 800 m). In D sites, the number of moss species was higher in the upper part of the gradient, although no clear pattern is shown. Liverworts, however, showed a mid-elevation peak with higher species number at elevations corresponding to potential areas of laurel forests.

**Fig 4:**
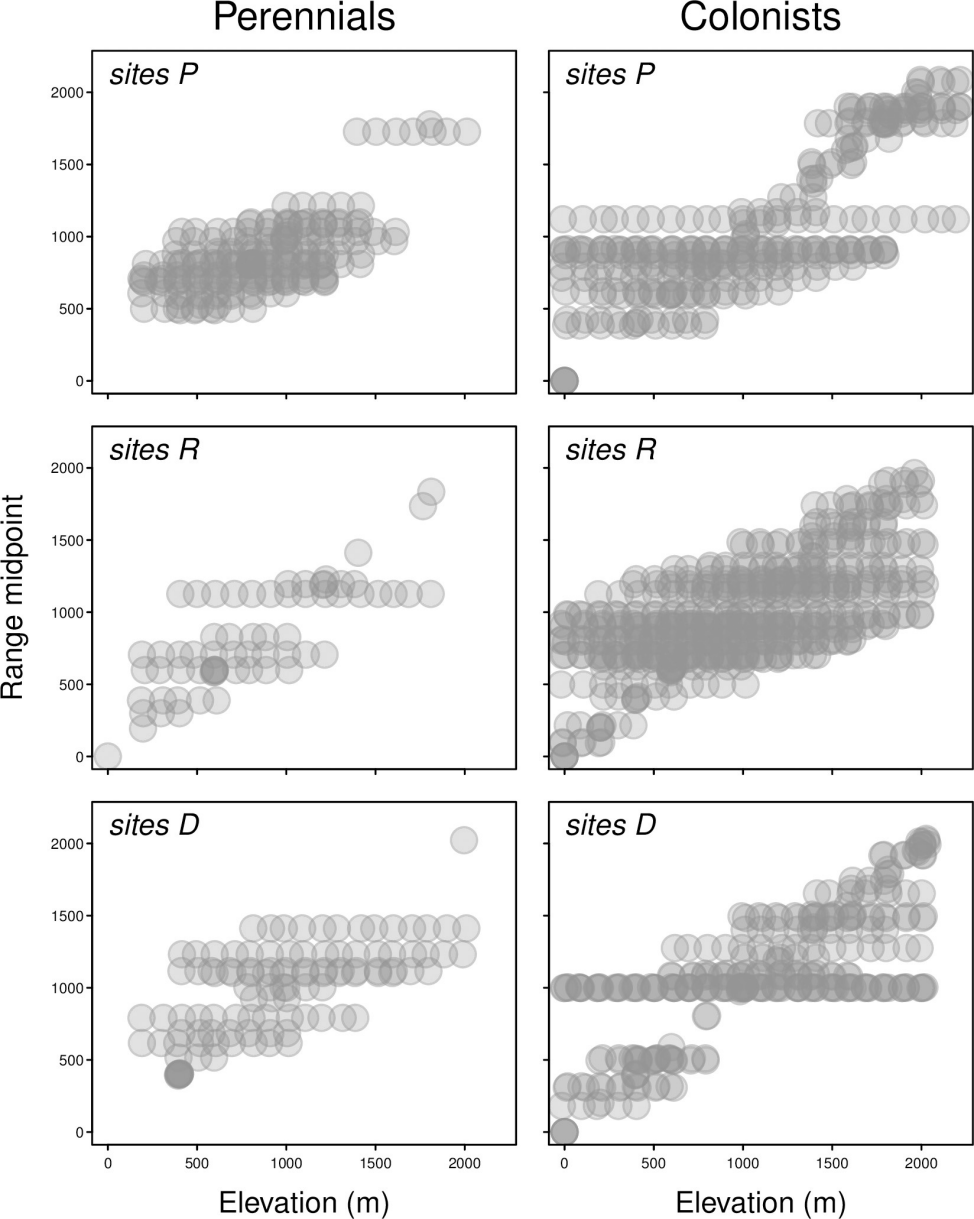
Patterns of elevational ranges for colonists and perennials along the three sites. Each dot represents the presence of a species within each 200 m band, each species gets as many dots as elevational steps between its upper- and lower recorded elevation. For clarity, dots were given transparency and a little jitter to depict local clustering. In this way, elevational ranges are depicted for colonists and perennials along the respective sites (P, R, and D).

Life strategy patterns along the elevational gradient also showed strong differences among all three sites types (Fig 3). A stronger shift was found regarding colonists and perennials between P and R sites. In P sites colonists were dominant in the lower and upper part of the gradient, while at mid-elevations, perennials (together with long-lived shuttles) became more species-rich. The opposite pattern was found in R sites, in which perennials at mid-elevations decreased in favour of colonists, which showed a uniform pattern along the whole gradient. A closer look at these life forms revealed that the proportion of perennial species is remarkably less in D and R sites than P sites, similarly, their elevational range is also reduced, while colonists follow the opposite pattern (Fig 4). Long-lived shuttles strongly decreased in potential areas of laurel forest between 200 m and 1200 m (Fig 3). In R sites, annual species showed a hump-shaped pattern, but they were virtually absent in the other two sites. For D sites there was again no clear pattern for life strategies, and colonists were the most common category along the entire transect.

If we consider substrate affinities then differences in the elevational distribution of substrates need to be accounted for between the three types of sites (Fig 3). In P sites, epiphytes were an important group, and most species occupying this substrate were present between 400 and 1200 m a.s.l. (corresponding to the well preserved laurel forest). In the same way, species growing on decaying wood were restricted to the mid-elevation plots, while terrestrials and rupicolous species were more homogeneously distributed along the elevational gradient. In P sites species are able to colonize different substrates. On the contrary, this was not found for species in the R and D sites, indicating species are restricted to one substrate. Most important change was the complete absence of epiphytes in R sites, in which bryophytes growing on soil, humus and rocks) were the most common group. In D sites epiphytes were present in mid-elevation plots, although less widespread, while rupicolous and terrestrial species were dominant throughout the transect.

**Fig 5:**
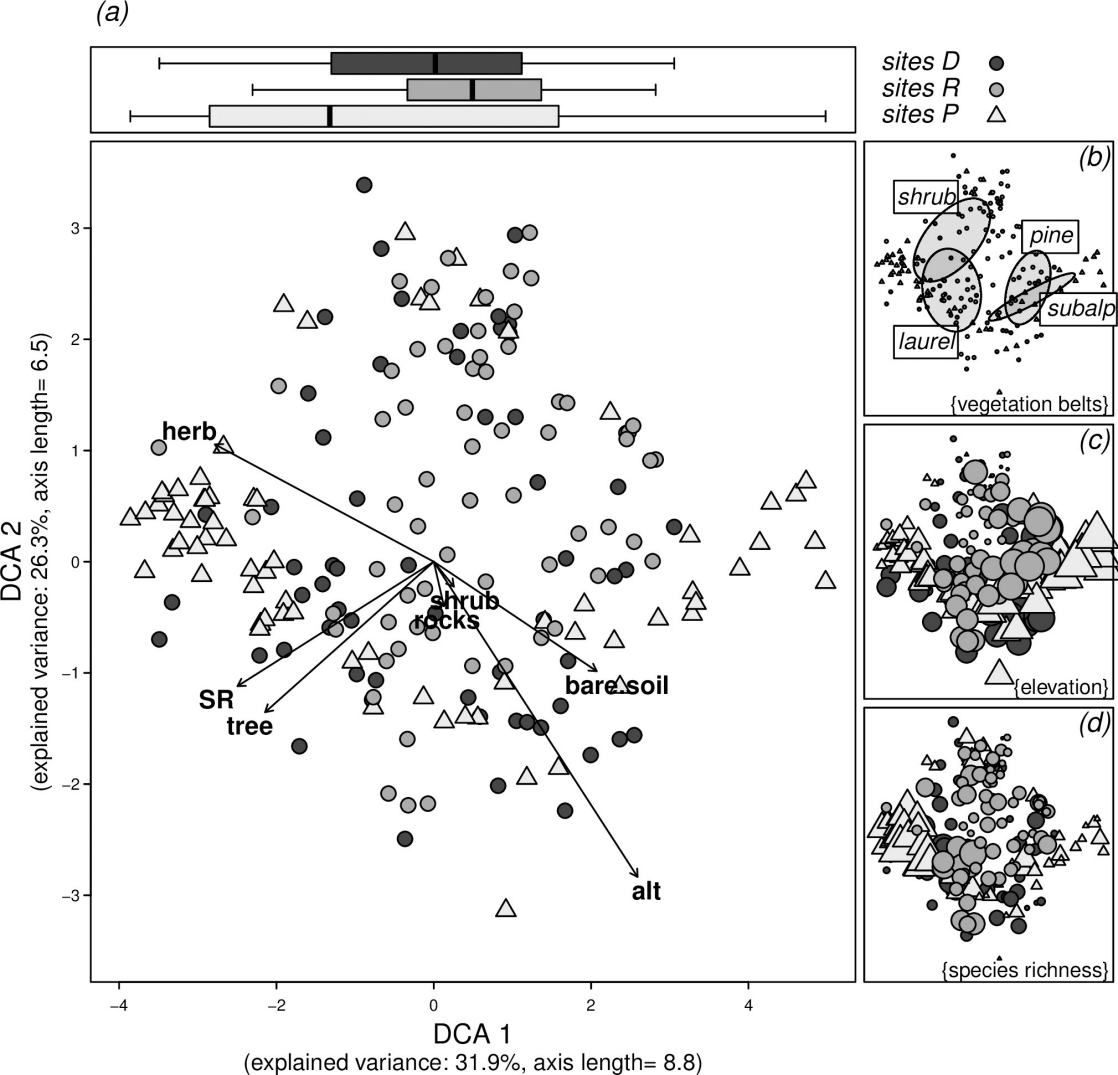
Ordination analysis (DCA) of subplots. (a) The main figure panel shows ordination positions of each subplot with different symbols for each site. Separation of subplots is high, indicated by axis length, and both axes together have an explained variance of 58.2%. Arrows indicate the score load of subplot characteristics (tree/shrub/herb/rocks: cover of trees, shrubs, herbs, and rocks; SR: species richness; soil: bare soil; alt: elevation). The boxplots above the main panel show the distribution of subplots along first axis, again separated for sites. The smaller panels on the right side highlight (b) the assignment of subplots to vegetation belts (centre of distribution indicated by ellipses), (c) elevation (symbol size according to elevation), and (d) species richness (symbol size according to species richness).

The first axis of a DCA ordination plot (Fig 5A) showed a higher heterogeneity of the P sites, indicating higher ß-diversity, which was also observed on the boxplot graph. R sites showed lower variability and thus lower ß-diversity. Separation of the three types of sites, although significant, is not very strong (r^2^ = 0.09; p<0.001), however, vegetation belts are significantly and well separated (r^2^ = 0.47; p<0.001). Higher species richness (SR) was associated with high herbaceous and tree cover. On the contrary, elevation and percentage of bare soil were related to lower species richness (Fig 5A). Communities in laurel forest and shrub belts are well differentiated, whereas pine forest and subalpine belt communities are very similar (Fig 5B). Elevation was related to axis 1, where high-elevation samples are located in the right side of the graph and lower elevation samples in the left side (Fig 5C). The opposite pattern was found for species richness, showing that the highest number of species is found on plots at mid and low-elevations (Fig 5D).

Results obtained after a Indicator Species Analysis, showed differences in indicators species in each type of vegetation (elevational belt) for each site type (Table 3). Important differences were observed on the elevational levels corresponding to the laurel forest potential areas. On P sites, perennial species like *Isothecium myosuroides*, *Lejeunea mandonii*, *Marchesinia mackaii* or *Rhynchostegium megapolitanum*, were indicators for this type of vegetation (Table 3). On R sites, however, indicator species were annual species (*Anthoceros caucasicus and Phaeoceros laevis*). No species was indicator of the laurel forest potential areas on D sites.

**Table 3:** Results obtained from the indicator species analysis for vegetation belts considered and for each site (P: Preserved sites, R: Roadside sites, D: Disturbed sites).

		Species	Moss / Liverwort	Life Strategy	Indicator value
Lowland vegetation potential areas					
	Site P	*Frullania ericoides*	L	L	0.5892
		*Tortella inflexa*	M	C	0.2168
	Site R	*Plagiochasma rupestre*	L	S	0.4567
		*Leptophascum leptophyllum*	M	C	0.3769
		*Tortula muralis*	M	C	0.3298
		*Ptychostomum imbricatulum*	M	C	0.2777
	Site D	*Tortella flavovirens*	M	C	0.5208
		*Tortella tortuosa*	M	C	0.3788
Laurel forest potential areas					
	Site P	*Lejeunea mandonii*	L	L	0.4969
		*Marchesinia mackaii*	L	P	0.3531
		*Heteroscyphus denticulatus*	L	P	0.3419
		*Frullania tamarisci*	L	L	0.3266
		*Rhynchostegium megapolitanum*	M	P	0.2804
		*Chiloscyphus coadunatus*	L	L	0.2667
		*Metzgeria furcata*	L	C	0.2652
		*Isothecium myosuroides*	M	P	0.2503
		*Thamnobryum alopecurum*	M	P	0.1905
	Site R	*Phaeoceros laevis*	A*	A	0.2619
		*Anthoceros caucasicus*.	A*	A	0.2089
Pine forest potential areas					
	Site P	*Hedwigia stellata*	M	L	0.2040
	Site R	*Didymodon fallax*	M	C	0.4903
		*Ptychostomum pallescens*	M	C	0.3457
		*Anacolia webbii*	M	L	0.1685
	Site D	*Orthotrichum rupestre*	M	C	0.3559
		*Dryptodon trichophyllus*	M	C	0.3058
Subalpine vegetation potential areas					
	Site P	*Grimmia montana*	M	C	0.4361
		*Pohlia cruda*	M	C	0.3626
		*Schistidium flaccidum*	M	C	0.2727
		*Oxyrhynchium schleicheri*	M	P	0.2613
	Site R	*Grimmia meridionalis*	M	C	0.4846
		*Syntrichia montana*	M	C	0.3730
		*Polytrichum juniperinum*	M	C	0.2127
	Site D	*Tortula inermis*	M	C	0.8000

Only species with significant indicator value are shown (*p*-value < 0.05). A*: Species belonging to division Anthocerotophyta, but included together with Marchantiophyta for the analysis. Life strategies are: P: Perennial; L: Long lived shuttle; S: Short lived shuttle; C: Colonist; A: Annual.

## Discussion

Our results showed that the hump shaped pattern obtained along the well preserved elevational gradient disappears in the case of both types of disturbed sites. Total species number did not differ among studied sites, but species composition and life strategies showed a clear response to the disturbance of mature communities. Specifically, certain groups, strictly depending on undisturbed shady regimes in natural forests (e.g., liverworts and perennial species, [42]) are the first to disappear, especially when the canopy is opened and microhabitats lose balanced humid and shady conditions [43]. Changes in species composition after different land use changes depending on light exposure has been also observed for bryophytes [44] and vascular plants [45]. With these trends, colonist species are able to expand their (elevational and thus ecological) distributional ranges as narrow-ranged species as *Exsertotheca intermedia*, *Homalothecium mandonii or Leptodon longisetus*, which are also macaronesian endemics, decreases (see S1 Table).

Bryophytes in our study sites showed different patterns of species richness with elevation. On the sites where preserved vegetation was studied (sites P), bryophyte species richness showed a marked hump-shaped relationship with elevation, as has been also observed for bryophytes in other islands [20,33,46] and also in continental settings [14,47,48]. However, this markedly unimodal pattern changes when considering sites affected by human activities. In this case we found a slightly hump-shaped pattern on sites that includes forest tracks (R) and no clear pattern at all on the disturbed sites (D) corresponding to silvicultural practice areas.

Major land-use changes have already occurred in the areas with highest biodiversity [8]. In this case, our richest ecosystem, the laurel forest, is the most sensitive to both types of human impact analysed. The dense evergreen canopy of the preserved laurel forest prevents most light from penetrating to the ground [49]. This canopy cover provides optimal conditions for species sensitive to environmental changes and alterations of their habitat, so that they are specific and restricted to these forests. Those changes are not so strong at lower and upper elevations, where canopy cover is low even in preserved sites.

The loss of the hump-shaped pattern in D and R sites may be attributable to the dominance of cosmopolitan colonist species on those two treatments. Many of these species are more tolerant to disturbed open habitats, so they can colonize open or modified habitats in a wide elevational range [50]. Colonist species are much more dependent on light exposure conditions than on temperature and humidity [37,51,52], and also of the presence of unstable habitats [37,51]. Thus, the removal of the forest canopy in both types of the altered sites favours colonists, which are generally more widespread. This in turn results in more homogeneous species assemblages along all the elevational belts. In contrast, perennials are very abundant on preserved sites, especially at mid-elevations, corresponding with well-preserved laurel forests. On disturbed and roadside sites, with the destruction of the forest, this optimal habitat for perennials disappears, and so do most of them, thus losing a great number of species at mid-elevations. In the altered sites, we can still find some perennial species as is the case of *Plasteurhynchium meridionale* (S1 Table), a characteristic epiphytic species in preserved sites that, however, can survive in rupicolous microhabitats in disturbed areas. This case shows complication in usage of bryophytes as bioindicators after disturbance. There is a delay in their reaction to habitat change, because of their ability to survive in microhabitats after the destruction of their optimal habitat [53]. Mosses are more abundant than liverworts on the two types of degraded sites along the whole gradient, while on preserved sites liverworts are dominant at mid-elevation contributing to the maximum of species richness at the middle of the transect. Liverworts are known to be a group that is highly dependent on moisture conditions (e.g. [42,50]). With the increase of light exposure and decrease in humidity after the disturbances the number of liverworts is lower than in preserved sites.

One important result of this study is the fact that epiphytes are not present in R sites, and drastically decrease on D sites. Epiphytes in P sites (especially at mid elevation belts) are mostly perennial pleurocarpic mosses and liverworts. The main factor driving this decrease (or the loss in the case of sites R) of the number of epiphytic species in both disturbed sites is the opening of the canopy and removal of mature trees. Many of these epiphytic species exhibit a preference for a given phorophyte [54,55]. The removal of the original forest is then unfavourable for epiphytes, even if new trees are planted (as is the case in cultivated stands on D sites) or other host species are present (as shrubby species growing on road margins on P sites).

### Conclusions

The observed differences in distribution patterns along the gradients highlight the importance of taking into consideration human disturbances when assessing biodiversity patterns, as was pointed out by Nogués-Bravo et al. 2008 [9]. General studies about biotic processes along elevational gradients might be underestimating human impacts, at least in historically disturbed islands, like La Palma. We may conclude that human impact strongly changes the elevational pattern of diversity, and these changes differ depending on the ecological and taxonomical group considered.

In fact, our results show that even in a group with few endemic species, as the bryophytes, the loss of endemics is one of the most important consequences of historical disturbances. In this sense, endemic macaronesian endemic species like *Homalothecium mandonii*, *Leptodon longisetus*, or *Exsertotheca intermedia* are indicators of the losses on quality of the bryophyte community. Strong change in community composition after disturbance have been found in elevations corresponding with laurel forests, thus we suggest that the remains of these forests should be of high conservation priority, in order to preserve bryophyte species richness and the uniqueness of these areas.

## Supporting information

S1 Table List of species recorded in the study plots and occurrence (number of microplots in which the species was present) in each site (P: preserved, R: roadside and D: disturbed). (XLSX)
